# Intramural Hematoma Causing Hematochezia After Colonoscopy With Polypectomy

**DOI:** 10.14309/crj.0000000000000129

**Published:** 2019-07-11

**Authors:** Aleksandar Gavrić, Rok Dežman, Sebastian Stefanović, Jan Drnovšek, Borut Štabuc

**Affiliations:** 1 Department of Gastroenterology and Hepatology, University Medical Center Ljubljana, Ljubljana, Slovenia; 2 Institute of Radiology, University Medical Centre Ljubljana, Ljubljana, Slovenia

## Abstract

Intramural hematoma of the colon is a rare complication of colonoscopy. We present a case of a 78-year-old woman on warfarin who presented with hematochezia and hypotension due to intramural hematoma of the sigmoid colon after colonoscopy with polypectomy of small polyps in the right colon.

## INTRODUCTION

Intramural hematoma of the gastrointestinal (GI) tract is rare and is least common in the colon.^1^ The main causes are trauma, anticoagulation therapy, and hematologic disorders. Spontaneous hematomas of unknown etiology have also been described.^1–4^ Only a few cases after diagnostic lower GI endoscopy without or with polypectomy have been published.^5–9^ Antithrombotic therapy and a redundant sigmoid colon, which often results in a difficult endoscopic procedure, looping, and requirements for increased external abdominal pressure are the main risk factors for a colonic hematoma after colonoscopy.^1,5^ Patients most commonly present with abdominal pain and hematochezia.^10^ Diagnosis is confirmed with colonoscopy and abdominal contrast-enhanced computed tomography.^10^ Initially, conservative therapy is indicated, but when symptoms persist, surgical resection is required.^10^ A fatal clinical outcome is rare.^9^

## CASE REPORT

A 78-year-old woman with a medical history of congestive heart failure, pacemaker implantation due to iatrogenic atrioventricular block after heart surgery, anticoagulant therapy with warfarin for a mechanical aortic and mitral valve, chronic kidney disease stage III, arterial hypertension, and with no history of abdominal surgery was admitted due to hematochezia that had started a few hours earlier. Three days previously, she had a colonoscopy for a further diagnostic workup of microcytic anemia. The endoscopic procedure was difficult due to a redundant sigmoid colon. As a result, increased external abdominal pressure was applied to reach the cecum. The procedure was prolonged; the total procedural time was 75 minutes. CO_2_ was used for insufflation.

During the withdrawal, 2 sessile polyps 8 and 3 mm in the cecum were resected with a cold snare. Histology revealed low-grade dysplasia adenomas. On physical examination, she appeared in mild distress. Digital rectal examination revealed hematochezia, and she was hypotensive. On examination, her abdomen was soft and non-tender. Hemoglobin was 7.9 g/dL, platelets were 255,000 μ/L, and the international normalized ratio was 1.05. Baseline hemoglobin was 9.6 g/dL. The patient received the last dose of low–molecular-weight heparin (LMWH) dalteparin 16 hours before diagnostic colonoscopy, which was reintroduced in the same evening after the procedure and continued in full dose until she was admitted to the hospital.

**Figure 1.**
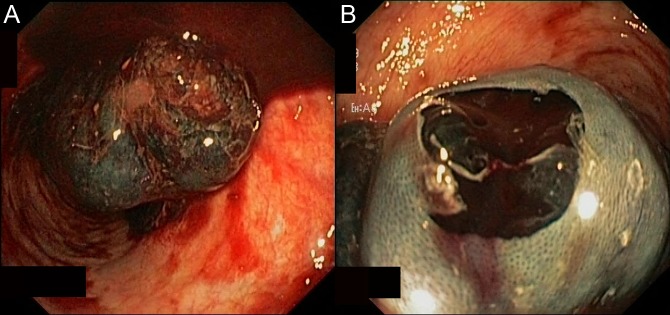
(A) Emergent colonoscopy showing dark-red lumen-occupying mass in the sigmoid colon, starting 10 cm from the anal verge. (B) Hematoma is clearly visible after a small piece of normal-appearing overlying mucosa was removed with biopsy forceps.

**Figure 2.**
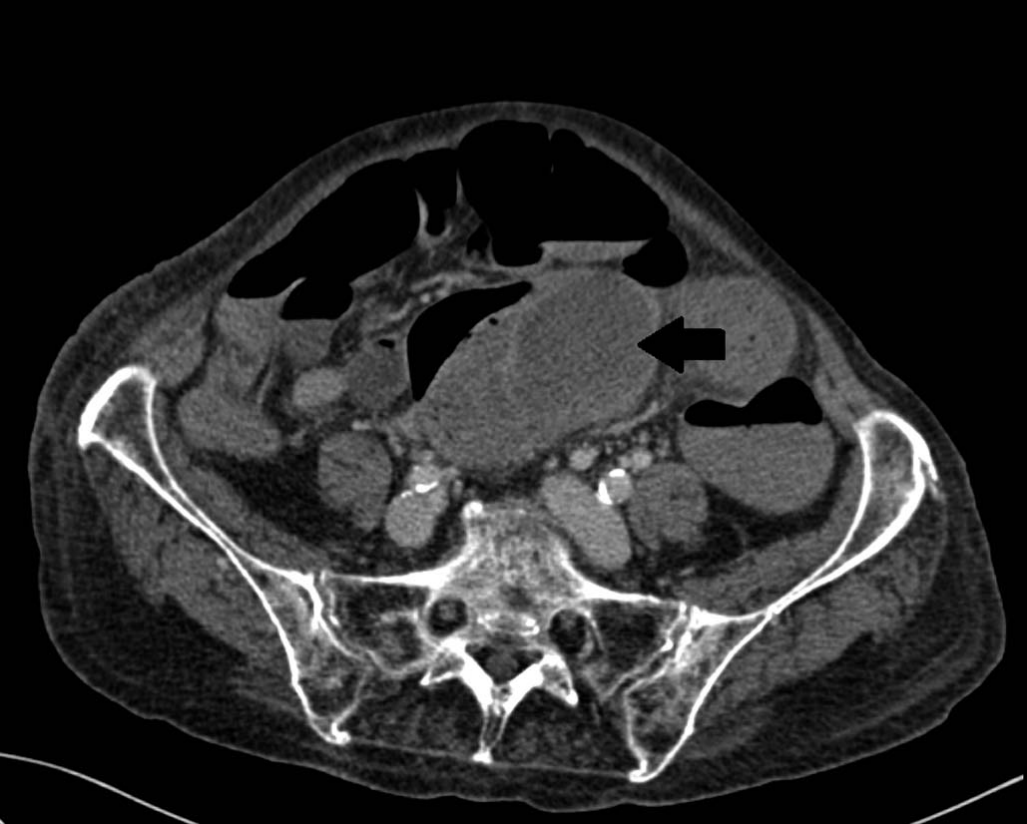
Axial contrast-enhanced computed tomography depicting an intramural hematoma of the sigmoid colon of 70 × 45 × 50 mm and hyperdense material in the lumen consistent with hemorrhagic content.

On arrival, post-polypectomy bleeding was suspected and an emergent colonoscopy was performed (Figure 1). She received 1 unit of packed red blood cells before the endoscopy, and 0.9% saline was started before the procedure. During the colonoscopy, a dark-red lumen-occupying mass was observed approximately 10 cm from the anal verge. The procedure was halted at 40 cm because of inadequate visualization of the lumen. She was admitted to the intensive care unit where she received an additional unit of packed red blood cells. After the transfusions, the patient was hemodynamically stable. We performed abdominal contrast-enhanced computed tomography (Figure 2). An elongated sigmoid colon filled with hemorrhagic content and a hypodense mass in the wall of the sigmoid colon consistent with the appearance of hematoma were identified. No proximal dilatation of the colon was present. Anticoagulation therapy was temporarily halted and was reintroduced 3 days after the emergent colonoscopy.

**Figure 3.**
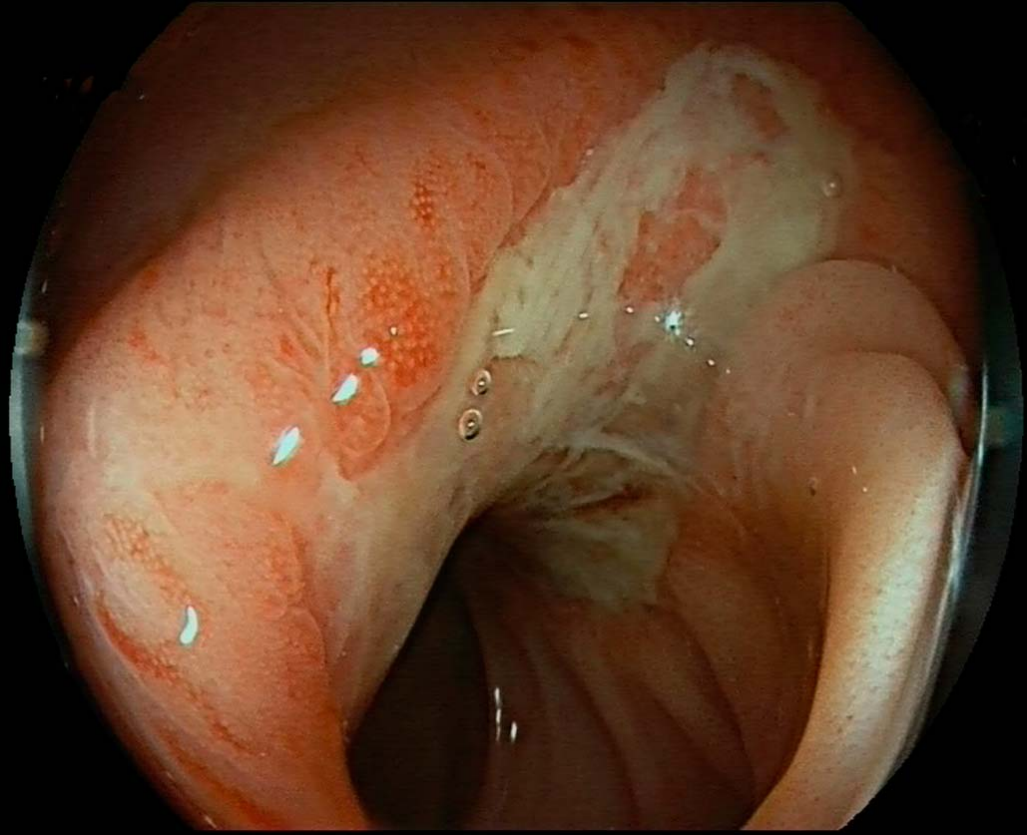
Control colonoscopy 9 days after the admission showing resolution of the hematoma.

During the hospitalization, the patient had several minor self-limiting episodes of hematochezia. After 9 days, we performed a control sigmoidoscopy (Figure 3). Erythema and ulcerations of the mucosa with no signs of hematoma were noted at the site of the previously seen hematoma. Ulcerations were noted 10 cm from the anal verge and extended up to 45 cm. Because of the stable clinical picture and low-risk histology of completely resected adenomas, only the left colon was examined. Our patient received 5 transfusions in total and was discharged home.

## DISCUSSION

Hematomas have been reported to occur in all parts of the GI tract but are least common in the colon.^1,11^ The estimated incidence of GI hematomas in anticoagulated patients is 1 in 250,000.^4^ The main causes are trauma, anticoagulation therapy, and hematologic disorders. Spontaneous hematomas of unknown etiology have also been described in the literature.^1–4^ Only a few cases after diagnostic lower GI endoscopy without or with polypectomy have been published.^5–9^ The common feature in almost all described cases is a difficult and long colonoscopy due to a redundant colon.

We assume that sigmoid hematoma in our case was caused by the colonoscope shaft in combination with prolonged and increased external abdominal pressure. In cases of intramural hematoma after diagnostic colonoscopy without polypectomy, the patients noticed hematochezia 6 and 12 hours after the procedure, and in one case, hematoma was noticed during the same colonoscopy on withdrawal.^7^ Our patient presented with hematochezia 72 hours after the colonoscopy. Unlike in other described cases, abdominal pain was not present in our case. In most cases, the clinical picture improved after the correction of anemia with transfusions and hemodynamic resuscitation, which are the cornerstone of conservative management of intramural colon hematoma.^6^ Our patient was hospitalized for 2 weeks.

The main reason for the prolonged hospitalization was several episodes of self-limiting hematochezia with the need for further transfusions and the need to monitor reintroduction of LMWH. In addition, our patient had a high risk of stroke and cardiovascular complications (calculated CHA_2_DS_2_-VASc score was at least 5) and a high risk of thrombosis if left without anticoagulant therapy. Surgical therapy is required when symptoms persist or the clinical picture deteriorates.^1,3,9^ The outcome of colon intramural hematoma is rarely fatal.^9^ A similarity between our case and those published in the literature is that more than half of the patients with intramural hematoma were taking antithrombotic agents.^2,4,6,7,9^

The management of antithrombotic therapy before initial colonoscopy in our patient was in accordance with the recommendations of the American Society for Gastrointestinal Society and European Society for Gastrointestinal Endoscopy, which state that warfarin should be stopped 5 days before endoscopy in high-risk conditions (prosthetic mitral valve in our case) and bridged with LMWH.^12,13^ The last dose of LMWH should be given ≥24 hours before colonoscopy and restarted in the evening of the same day after colonoscopy. Our case deviated from the recommendations only in discontinuing LMWH approximately 16 hours before colonoscopy. In conclusion, even though a rare diagnosis, intramural hematoma must be suspected in all anticoagulated patients with hematochezia and abdominal pain after colonoscopy, especially in difficult cases in which increased external abdominal pressure was required to reach the cecum.

## DISCLOSURES

Author contributions: A. Gavrić wrote and reviewed the manuscript. R. Dežman reviewed the manuscript and provided and commented CT images. S. Stefanović, J. Drnovšek, and B. Štabuc reviewed the manuscript. A. Gavrić is the article guarantor.

Financial disclosure: None to report.

Informed consent was obtained for this case report.
